# Comparison of the performance of ITS1 and ITS2 as barcodes in amplicon-based sequencing of bioaerosols

**DOI:** 10.7717/peerj.8523

**Published:** 2020-02-17

**Authors:** Hamza Mbareche, Marc Veillette, Guillaume Bilodeau, Caroline Duchaine

**Affiliations:** 1 Institut Universitaire de Cardiologie et de Pneumologie de Québec, Quebec City, Quebec, Canada; 2 Canadian Food Inspection Agency, Pathogen Identification Research Lab, Ottawa, Canada; 3 Département de biochimie, de microbiologie et de bio-informatique, Faculté des sciences et de génie, Université Laval, Quebec City, Canada

**Keywords:** Bioaerosols, ITS1, ITS2, High-throughput sequencing, Fungi, Barcodes

## Abstract

This paper presents the performance of two eukaryotic genomic ribosomal regions, ITS1 and ITS2, in describing fungal diversity in aerosol samples using amplicon-based High-Throughput Sequencing (HTS). Composting sites, biomethanization facilities, and dairy farms, all affected by the presence of fungi, were visited to collect air samples. The amplicon-based HTS approach is a target enrichment method that relies on the amplification of a specific target using particular primers before sequencing. Thus, the results are highly dependent on the quality of amplification. For this reason, the authors of this paper used a shotgun metagenomic approach to compare its outcome with the amplicon-based method. Indeed, shotgun metagenomic does not rely on any amplification prior to sequencing, because all genes are sequenced without a specific target. In addition, culture methods were added to the analyses in biomethanization and dairy farms samples to validate their contribution to fungal diversity of aerosols. The results obtained are unequivocal towards ITS1 outperformance to ITS2 in terms of richness, and taxonomic coverage. The differential abundance analysis did demonstrate that some taxa were exclusively detected only by ITS2, and vice-versa for ITS1. However, the shotgun metagenomic approach showed a taxonomic profile more resembling to ITS1 than ITS2. Based on these results, neither of the barcodes evaluated is perfect in terms of distinguishing all species. Using both barcodes offers a broader view of the fungal aerosol population. However, with the actual knowledge, the authors strongly recommend using ITS1 as a universal fungal barcode for quick general analyses of diversity and when limited financial resources are available, primarily due its ability to capture taxonomic profiles similar to those obtained using the shotgun metagenomic. The culture comparison with amplicon-based sequencing showed the complementarity of both approaches in describing the most abundant taxa.

## Introduction

Natural air contains a class of particulate matter of biological origin referred to as bioaerosols. Bioaerosols include: living and dead fungi and bacteria, viruses, bacterial endotoxins, mycotoxins, *β* (1, 3)-glucans, pollens and other airborne allergens, etc. (Macher et al., 1999; Douwes et al., 2003; Després et al., 2012). Both natural and artificial (manmade), bioaerosols are ubiquitous, highly variable and complex to identify, mainly because they can originate from various sources (e.g., plants, soil, water, humans; Paez-Rubio et al., 2005; Taha et al., 2006; Hospodsky et al., 2012). Their composition depends on the source, aerosolization mechanisms and environmental conditions (Foarde et al., 1993; Pasanen, Pasanen & Janunen, 2000; Pillai & Ricke, 2002; Jones & Harrison, 2004; Bonifait et al., 2017; Mbareche et al., 2017). The dispersal of bioaerosols can have major impacts on public health through their effects from inhalation and potential ingestion. The inhalable particles can reach deep parts of the respiratory system, causing a wide range of acute and chronic diseases such as allergies, asthma, rhinitis, sinusitis and bronchitis. Bioaerosols are also involved in adverse health effects caused by some occupational exposures (Brown & Hovmøller, 2002; Douwes et al., 2003; Brodie et al., 2007; Eduard et al., 2012; Heederik & Von Mutius, 2012), as well as the dispersal and transmission of infectious diseases agents (Roy & Milton, 2004; Yu et al., 2004; Eames et al., 2009; Li et al., 2007).

Fungal spores are ubiquitous in the air and their diversity and/or concentration vary depending on the climate, geographical conditions and the presence of fungal growth sources in the environment (Ruzer & Harley, 2005; Kakde, 2012). Industrial activities that are mainly linked to agriculture have some of the highest rates of fungal exposure in their environments. These conditions are created by the presence of decaying materials such as hay, peat, wood dust, manure, biosolids, and organic wastes, like compost (Gilbert & Duchaine, 2009). Dairy farms and waste treatment sites are examples of such environments where humans are exposed to a wide variety of fungi (Mbareche et al., 2017; Mbareche et al., 2018a; Mbareche et al., 2019a). Fungi can also become airborne in environments where they are deliberately introduced, such as in the food industry where fungi are used for production (Morell et al., 2011; Simon & Duquenne, 2014). The health effects of fungal exposure range from relatively serious effects such as allergy-related diseases, pulmonary inflammation, increased sensitivity to endotoxins, and pulmonary embolisms to milder effects such as irritation of the nose and eyes, bronchial irritation, mucous membrane irritation syndrome, nasal congestion, and sore throat (Wyngaarden, Smith & Bennett, 1992; Fogelmark, Sjostrand & Rylander, 1994; Rylander, 1996; Burge & Rogers, 2000; Arshad et al., 2001; Hardin, Kelman & Saxon, 2003; Daisey, Angell & Apte, 2003; Pieckova & Wilkins, 2004; Stark et al., 2005; Zekovic et al., 2005; Beezhold et al., 2008; Bush, 2008; Porter et al., 2009; Sarkar et al., 2010; Selman et al., 2010; Glass & Amedee, 2011; Chowdhary et al., 2014). Exposure to a variety of fungi can also result in infections, especially in people with impaired immune systems (Latgé, 1999; Nucci & Anaissie, 2007; Rodriguez & Ramos, 2014; Velegraki et al., 2015). However, the impact of fungi on occupational health could be still largely underestimated. The numerous fungi that are still undocumented present a barrier to establishing a clear link between respiratory problems and fungal exposure (Bush et al., 2006; Tischer & Heinrich, 2013).

Historically, fungi have been identified based on the morphological characteristics of pure cultures in agar media. This process has also been used in more recent exposure studies (Sanchez-Monedero & Stentiford, 2003; Taha et al., 2006; Schlosser et al., 2012; Park et al., 2013; Ghiasian, Maghood & Aghamirian, 2017; Liu et al., 2018). However, most fungal species are difficult to isolate using common culture methods. Therefore, using these techniques may lead to an underestimation of the fungal diversity in bioaerosols, especially considering that the kingdom of Fungi is one of the most diverse (Blackwell, 2011). Molecular methods are offering new perspectives on the occurrence and the ecological impact of microbes. One of these methods includes high-throughput sequencing (HTS), which offers a more thorough analysis of the microbial content of a sample because of the millions of sequences that are generated, and that it detects fungal DNA independent of their culturability and viability. Using the appropriate bioinformatics tools, this technology can characterize thousands of species, referred to as OTUs (operational taxonomic units), from environmental samples. The success of the amplicon-based HTS approach resides in the critical decision of which DNA region to use as the barcode. These universal phylogenetic markers are selected based on a number of criteria, including their ubiquitous presence across taxa and having sufficient sequence variation between taxa. While using the small ribosomal DNA (rDNA) subunit 16S is the obvious choice for prokaryotes, eukaryotic species present more challenges for the metabarcoding community. For example, the mitochondrial gene COI has been used as a universal barcode for animals (Hebert, Ratnasingham & deWaard, 2003). The combination of the rbcl and matK genes has been proposed as the universal plant barcode (Hollingsworth & Forrest, 2009). The *internal transcribed spacer* (ITS) region of rDNA is the most used barcode to study fungal diversity (Roe et al., 2010; Dentinger, Didukh & Moncalvo, 2011; Schoch et al., 2012). ITS region contains three partitions: ITS1, 5.8S and ITS2. The length of ITS sequence is highly variable from one fungal species to another and it is strongly dependent on the primers used to target the DNA sequence. For example, in Ascomycota and Basidiomycota the sequence lengths range between 600 and 900 bp (Toju et al., 2012). Amplicon-based HTS approaches involve an enrichment step prior to sequencing, which involves using PCR amplification of the targeted barcode. The most commonly used high-throughput sequencers have a maximum read length limitation (MiSeq Illumina –2 ×300 bp; HiSeq 2500 Illumina –up to 2 ×125 bp; Ion PGM –400 bp). This limitation forces the use of only one of the two sub regions (ITS1 or ITS2) when applying the amplicon-based HTS approach for determining fungal diversity. The 5.8S region does not contain a sufficient number of informative sites that can be used for phylogenetic studies and DNA barcoding (Khot, Ko & Fredricks, 2014). Studies using environmental samples from soil, mangroves, plants and aquatic ecosystems or retrieved ITS sequences from GenBank (using ‘internal transcribed spacer’ as the keyword) gave mixed reviews on the performances of ITS1 and ITS2 in documenting and characterizing fungal biodiversity (Kelly et al., 2011; Arfi et al., 2012; Ihrmark et al., 2012; Heinrichs, De Hoog & Haase, 2012; Osmundson et al., 2013; Blaalid et al., 2013; Bazzicalupo, Bàlint & Schmitt, 2013; Monard, Gantner & Stenild, 2013; Kohout et al., 2014; Op De Beeck et al., 2014; Wang et al., 2014; Ishii, Ishida & Kagami, 2015; Tedersoo et al., 2015a; Tedersoo et al., 2015b). Comparing studies on fungal diversity from different scientific fields is challenging, difficult, and nearly impossible, due to their use of different barcodes and methods of analyses.

Another HTS approach, referred to as shotgun metagenomics, consists of the untargeted sequencing of all microbial genomes in a sample (Quince et al., 2017). Shotgun sequencing is not subject to amplicon length limitations or the PCR biases imposed by HTS approaches based on ITS1 or ITS2. However, shotgun sequencing presents many other known limitations. The most important one is that there can be a low relative proportion of ribosomal DNA from the metagenomes of the microbes of interest (fungi in this case) compared to the pool of genes that are present in samples. This may complicate the detection of fungal species when there is no enrichment.

This large-scale study uses air samples from waste treatment sites and dairy farms to systematically compare the performance of ITS1 and ITS2 in metabarcoding analyses of fungal diversity in aerosols. The study was designed as a result of discussions about which amplicon-based HTS approaches would best describe aerosol fungal exposure. The analyses include sequence length distribution, richness and diversity indices, multivariate analyses, differential abundance, species discrimination efficiency and taxonomy analyses. In addition, shotgun metagenomics was applied to air samples from dairy farms in order to compare its results with the HTS approach based on ITS1 and ITS2. Furthermore, diversity was measured using the culture method to evaluate its contribution to fungal bioaerosol studies. This work provides new insights into the use of both ITS subregions in order to assess fungal aerosol populations and also provides a guide for which strategies to use for analyzing particular taxonomic groups. The results obtained suggest that neither of the barcodes evaluated is perfect for distinguishing all species. We recommend using ITS1 mainly because it performed better than ITS2 in describing the fungal diversity of bioaerosols. The culture comparison with amplicon-based sequencing showed the complementarity of both approaches in describing the most abundant taxa.

## Materials and Methods

### Description of the environmental conditions of field sampling

#### Compost

In 2014–2015, two different composting plants located in the province of Quebec, Canada were visited during a year-long sampling schedule to monitor the composting processes. The composting plants treat two different types of raw materials: household green waste (domestic) and pig carcasses and placenta (animal). In total, 50 samples were collected in the composting sites during the four seasons. Detailed information about the sampling schedule and conditions are presented in the original composting study report (Bonifait et al., 2017).

#### Biomethanization

Samples were collected from two different biomethanization facilities during the summer of 2015 and the winter of 2016. As previously described in Mbareche et al. (2018a); Mbareche et al. (2018b), the first facility (BF1) treats primary and secondary sludge from wastewater treatment plants, and industrial waste. The second facility (BF2) treats domestic municipal waste. In total, 32 samples were collected in the biomethanization facilities during summer and winter. Detailed information about the sampling sites and conditions can be found in the original study report (Dubuis et al., 2017).

#### Dairy farms

As previously described in Mbareche et al. (2019a), air was collected from five dairy farms in Eastern Canada during the summer of 2016. At each farm, a sampling site was designated based on where activities that generate the most bioaerosols took place. Every farm building presented different characteristics like age, space, type of ventilation, number of animals, method of milking and type of food given to animals. Detailed information about the sampling sites and conditions can be found in the original study report (Mbareche et al., 2019a).

### Air sampling

Air samples were collected using a liquid cyclonic impactor Coriolis µ^®^ (Bertin Technologies, Montigny-le-Bretonneux, France). The sampler was set at 200 L/min for 10 min (2m^3^ of air per sample) and placed within 1–2 m of the bioaerosol source. The sampling sites were chosen according to workers’ activities. The vortex created by the airflow make the air particles impact in the liquid, which is a fifteen millilitres of phosphate buffer saline (PBS) solution with a concentration of 50 mM and a pH of 7.4.

### Culture-based approach to study fungal diversity

The following method was previously described in Mbareche et al. (2019a). Here is a summary: one millilitre of the 15 ml Coriolis sampling liquid was used to perform a serial dilution from 10^0^ to 10^−4^ concentration/ml. The dilutions were made using 0.9% saline and 0.1% Tween20 solution and were performed in triplicate. Tween20 is a detergent that makes spores less hydrophobic and easier to collect. One hundred microlitres of each triplicate were plated on Rose Bengal Agar with chloramphenicol at a concentration of 50 µg/ml. Half of the Petri dishes were incubated at 25 °C for mesophilic mould growth and the other half at 50 °C for thermophilic mould growth, specifically the fungus/mould *Aspergillus fumigatus*. After 5 days of incubation, the moulds were identified and the counts were translated into CFU/m^3^.

#### Identification of isolates

As previously described in Mbareche et al. (2019a), spores from cultured fungi were recovered in one millilitre of a 0.9% saline and 0.1% Tween20 solution and stored in an Eppendorf tube. Two hundred microlitres of the collection liquid were placed in an FTA card (sample collection cards; Qiagen, Mississauga, Ontario, Canada). Five punches from the spiked zone of the FTA card were placed in a microtube and washed three times with the FTA purification agent. The washing step is necessary as it removes the chemical substrates in the FTA card that may alter the subsequent amplification step. Forty-eight microlitres of the master mix solution (described in Supplemental Information 1) were placed in each microtube followed by the amplification and sequencing of ITS region. The protocol described by White and his collaborators (White et al., 1990) was performed at CHU (*Centre hospitalier de l’Université Laval*). The following oligonucleotides were used for ITS region amplification:

ITS1: 5′-TCCGTAGGTGAACCTGCGG-3′

ITS4: 5′-TCCTCCGCTTATTGATATGC-3′

The isolates were identified by comparing the sequences obtained with sequences in the UNITE 7.2 database with the BLASTn option.

### Concentration of fungal spores in aerosols

The following method was used because it is optimal for recovering fungal spores from air samples, as described in detail by Mbareche and his coauthors in 2019 (Mbareche et al., 2019b). Briefly, the liquid suspension from the Coriolis cone was filtered through a 2.5 cm polycarbonate membrane (0.2-mm pore size; Millipore) using a vacuum filtration unit. The filters were flash-frozen and pulverized using a tungsten steel bead in an Eppendorf tube in a bead-beating machine (a Mixer Mill MM301, Retsch, Düsseldorf, Germany). Aliquots of the liquid containing the pulverized filter particles were used for the first step of the DNA extraction procedure.

### DNA extraction

The extraction method was previously described in Mbareche et al. (2019a). In brief, using the same apparatus, bead-beating was performed a second time using glass beads at a frequency of 20 movements per second for 10 min to ensure that all of the cells were ruptured. The MoBio PowerLyser^®^ Powersoil^®^ Isolation DNA kit (Carlsbad, CA, U.S.A) was used to extract DNA from the samples following the manufacturer’s instructions. After elution, DNA was kept at −20 °C until future analyses.

### MiSeq Illumina sequencing

The amplification primers chosen for amplicon-based HTS were based on Tedersoo and his colleagues in their analyses of primer biases in fungal metabarcoding (Tedersoo et al., 2015a; Tedersoo et al., 2015b). As previously described in Mbareche et al., (2018a, 2018b, 2019a). Amplification of the amplicons, equimolar pooling and sequencing were performed at the *Plateforme d’analyses génomiques* (IBIS, Université Laval, Quebec City, Canada). Briefly, amplification of ITS regions was performed using the sequence-specific regions (ITS1 and ITS2) described by Tedersoo et al. (2015a); Tedersoo et al. (2015b) and references therein, using a two-step dual-indexed PCR approach specifically designed for Illumina instruments. First, the gene-specific sequence was fused to the Illumina TruSeq sequencing primers. Next, PCR was carried out on a total volume of 25 µL of liquid made up of: 1X Q5 buffer (NEB), 0.25 µM of each primer, 200 µM of each of the dNTPs, 1U of Q5 High-Fidelity DNA polymerase (NEB) and 1 µL of template cDNA. The PCR started with an initial denaturation at 98 °C for 30s followed by 35 cycles of denaturation at 98 °C for 10s, annealing at 55 °C for 10s, extension at 72 °C for 30s and a final extension step at 72 °C for 2 min. The PCR reaction was purified using an Axygen PCR cleanup kit (Axygen). The quality of the purified PCR products was verified with electrophoresis (1% agarose gel). A dilution of 50 to 100 fold of this purified product was used as a template for a second round of PCR with the goal of adding barcodes (dual-indexed) and the missing sequences required for Illumina sequencing. The conditions for the second round of PCR cycling were identical to the first PCR, but with 12 cycles. The PCR reactions were purified as above, checked for quality on a DNA7500 Bioanlayzer chip (Agilent) and then quantified spectrophotometrically with a Nanodrop 1000 (Thermo Fisher Scientific). Barcoded Amplicons were pooled in equimolar concentration for sequencing on the illumina Miseq. The primer sequences used for amplification are presented in Table 1.

**Table 1:** Primers used for amplification of ITS1 and ITS2 barcodes and for Illumina Miseq sequencing.

Primers name	Features	Sequence	Barcode	PCR
ITS1Fngs	Fwd, tagged	ACACTCTTTCCCTACACGACGCTCTTCCGATCTGGTCATTTAGAGGAAGTAA	ITS1	First
ITS2	Rev	GTGACTGGAGTTCAGACGTGTGCTCTTCCGATCTGCTGCGTTCTTCATCGATGC	ITS1	First
ITS3tagmix1	Fwd	ACACTCTTTCCCTACACGACGCTCTTCCGATCTTAGACTCGTCATCGATGAAGAACGCAG	ITS2	First
ITS3tagmix2	Fwd	ACACTCTTTCCCTACACGACGCTCTTCCGATCTTAGACTCGTCAACGATGAAGAACGCAG	ITS2	First
ITS3tagmix3	Fwd	ACACTCTTTCCCTACACGACGCTCTTCCGATCTTAGACTCGTCACCGATGAAGAACGCAG	ITS2	First
ITS3tagmix4	Fwd	ACACTCTTTCCCTACACGACGCTCTTCCGATCTTAGACTCGTCATCGATGAAGAACGTAG	ITS2	First
ITS3tagmix5	Fwd	ACACTCTTTCCCTACACGACGCTCTTCCGATCTTAGACTCGTCATCGATGAAGAACGTGG	ITS2	First
ITS4ngs	Rev	GTGACTGGAGTTCAGACGTGTGCTCTTCCGATCTTTCCTSCGCTTATTGATATGC	ITS2	First
Generic forward	Fwd	AATGATACGGCGACCACCGAGATCTACAC[index1]ACACTCTTTCCCTCACGAC	ITS1&2	Second
Generic reverse	Rev	CAAGCAGAAGACGGCATACGAGAT[index2]GTGACTGGAGTTCAGACGTGT.	ITS1&2	Second

**Notes.**

Please note that the primers used in this work contain Illumina specific sequences protected by intellectual property (Oligonucleotide sequences ©2007–2013 Illumina, Inc.  All rights reserved. Derivative works created by Illumina customers are authorized for use with Illumina instruments and products only. All other uses are strictly prohibited).

For the shotgun metagenomics, library preparation and sequencing was also performed at the *Plateforme d’analyses génomiques* (IBIS, Université Laval, Quebec, Canada). In brief, Genomic DNA (500 ng in 55 ul) was mechanically fragmented for 40 s using a covaris M220 (Covaris, Woburn MA, USA) with default settings. Fragmented DNA was transferred to PCR tubes and library synthesis was performed with the NEB Next Ultra II (New England Biolabs) according to the manufacturer’s instructions. TruSeq HT adapters (Illumina, SanDiego, CA, USA) were used to barcode the samples. The libraries were quantified and pooled using an equimolar ratio and sequenced on an Illumina MiSeq 300 base pair paired-end run (600 cycle, v3 kit).

### Bioinformatics

After demultiplexing the raw FASTQ files, the reads generated from the paired-end sequencing were paired and quality-filtered using MOTHUR 1.35.1 (Schloss et al., 2009). The quality-filtering consisted of discarding reads with ambiguous sequences, sequence length ranges from 100 bp to 600 bp, and maximum homopolymer lengths of 8. The quality filter was based on the Phred score with a minimum value of 33. Identical sequences were combined to reduce the time analyses. Next, sequences that occurred only once (singletons) were discarded. This dereplication step was performed using USEARCH 7.0.1090 (Edgar, 2010). The ITS1 and ITS2 fungal sequences were then extracted from the dataset with ITSx (Mistry et al., 2013; Bengtsson-Palme et al., 2013). Only the sequences belonging to the kingdom Fungi were kept for further analyses. For ITS1, less than 3% of sequences were not on fungal origin , whilst for ITS2 less than 5% of sequences were not fungal sequences. Operational taxonomic units (OTUs) with a 97% similarity cut-off were clustered using UPARSE 7.1 (Edgar, 2013). The similarity threshold (97%) is commonly used in OTU-based analyses and has been shown to be an optimal threshold when using ITS to identify fungi (Koljalg et al., 2013). The chimeric sequences were identified and removed with UNITE-UCHIME (Nilsson et al., 2015). QIIME 1.9.1 (Caporaso et al., 2010) was used for taxonomy assignment with UNITE 7.2 fungal ITS reference training data set. QIIME 1.9.1 was also used to generate an OTU table. Fungal diversity was analyzed by using several different QIIME scripts. The scripts used for alpha/beta diversity, multivariate analyses, differential abundance and taxonomy analyses are listed on the following website: http://qiime.org/scripts/.

For the shotgun metagenome sequences, samples were demultiplexed, quality-controlled and assembled for taxonomic profiling. This was done using the standard MEGAN6 pipeline (Huson et al., 2007) and the default MetaPhlan 2.0 analyses pipeline, which was run on the PyCharm CE platform for python 2.7 (Truong et al., 2015). After comparing the outcomes from both programs, the results obtained by MetaPhlan 2.0 were used in this work because of the flexibility of the command line tool. In addition, all the organisms’ ITS regions were extracted from the quality-controlled metagenome sequences using SORTMERNA v2.1 (Kopylova, Noé & Touzet, 2012). The extracted ITS sequences were classified against the UNITE 7.2 reference database for taxonomic identification.

The following are links to the bioinformatics protocols that were applied for shotgun metagenomic analyses:

MetaPhlAn2 pipeline: https://bitbucket.org/biobakery/metaphlan2

MEGAN6 tutorial: https://software-ab.informatik.uni-tuebingen.de/download/megan6/manual.pdf

Source code for SORTMERNA: https://github.com/biocore/sortmerna/releases/tag/2.1

### Statistical analyses

For alpha diversity measures, normality was verified by the D′ Agostino and Pearson omnibus normality test. As normality was not demonstrated, the non-parametric Mann–Whitney *U* test was used to assess the significance of the differences between ITS1 and ITS2 in air samples from compost, biomethanization and dairy farms. A *p*-value ≤ 0.05 was considered statistically significant. The results were analyzed using the software GraphPad Prism 5.03 (GraphPad Software, Inc.). The Bray-Curtis index was used for the pairwise comparison of the samples. The Bray-Curtis index values range between 0 and 1, where 0 means the two samples are identical and 1 means that they are completely different. The QIIME script for *beta diversity* analyses was used to produce the Bray-Curtis matrix, which includes information about OTU abundance. It is mandatory to use a rarefied OTU table for the Bray-Curtis calculation, because Bray-Curtis is based on absolute abundances of the OTUs. The same rarefaction depth from the rarefaction curves described previously was used for the multivariate analysis (40,000 sequences for compost and dairy farm samples and 9,000 sequences for biomethanization samples). Inter-sample distances were represented in a dimensional space using ordination. One of the most commonly used methods to evaluate ordination patterns is the principal coordinate analyses (PCoA). Coordinates are calculated from the dissimilarity matrix. After, the matrices were transformed to coordinates, the *principal coordinates* script from QIIME was used to produce the PCoA plots. For each environment, samples were separated according to the barcode used (ITS1 and ITS2) and the environmental factors that could explain community variation (composting sites: domestic and animal; biomethanization facilities: BF1 and BF2; dairy farms: DF1 to DF5). To validate statistically the clustering observed with the multivariate analyses (PCoA figures), we applied a PERMANOVA test. The *compare categories* QIIME script was used to generate the statistical results. PERMANOVA is a non-parametric test, and the significance was determined through permutations (999). A *p*-value ≤ 0.05 was considered statistically significant.

To identify species that had significantly different abundances depending on the barcode used, a statistical test designed specifically for the differential analyses of count data was used. Using this Mann–Whitney *U* test, OTU frequencies can be compared in groups of samples and whether or not the two groups of samples have statistically different OTU abundances can be ascertained. This test is non-parametric and uses absolute data counts rather than relative abundances. More specifically, the output of the test contains the test statistic, the *p*-value corrected for multiple comparisons and a mean count for each OTU in the given sample group. The Mann–Whitney *U* test was used following instructions from the *group significance* QIIME script. The non-parametric Mann–Whitney *U* test was used to ascertain whether or not the differences in OTU abundances are statistically significant between ITS1 and ITS2. To test OTU differential abundance, the null hypothesis was that the populations that the two groups of samples were collected from having equal means. The range of p-values obtained for the 50 most differentially abundant OTUs between ITS1 and ITS2 are presented in the differential abundance section of the results.

## Results

### Summary of sequencing data processing

Table 2 presents a summary of the sequencing information from the number of raw reads to the number of OTU clusters recorded at different steps of the bioinformatics data processing. Samples from composting sites (27), biomethanization facilities (16) and dairy farms (5) were compared based on the barcode used (ITS1 & ITS2). The number of raw reads from MiSeq sequencer was comparable when either one of the barcodes was used (same order of magnitude). The sequence lengths were different for ITS1 and ITS2. ITS2 sequences were systematically longer than ITS1 sequences in the three environments. The mean length of ITS1 sequences ranged from 278 bp to 294 bp and they ranged from 364 bp to 398 bp for ITS2 sequences. Unexpectedly, the mean length of ITS2 sequences was exactly the same (364 bp) across compost and biomethanization samples. After quality filtering, we excluded the singletons for subsequent analyses (Brown et al., 2015). In general, the percentage of singletons was the same for ITS1 and ITS2, except in compost samples. Singletons represented only 5.6% of the sequences when ITS1 was used compared to 14% for ITS2. At the end of data processing, clusters of OTUs were formed. The number of OTUs was two to five times higher when ITS1 was targeted compared to ITS2. The huge number of sequences lost in the process from raw sequences to post quality filtering is due to the stringent threshold values of quality used, like ambiguous sequences, too long or too short sequences, and sequences of bad quality were not allowed.

**Table 2:** Summary of the HTS data during the bioinformatics treatment process. ITS1 and ITS2 amplicons are compared in the bioaerosols from the three environments studies.

	Number of raw seq. from MiSeq platform		Mean length of seq. after paired-end assembly		Number of seq. after quality filtering (% of singletons)		Number of OTUs	
	**ITS1**	**ITS2**	**ITS1**	**ITS2**	**ITS1**	**ITS2**	**ITS1**	**ITS2**
Compost	3,871,313	3,680,926	294	364	44,438 (5.6%)	53,001 (14%)	1,208	772
Biomethanization	675,642	730,688	281	398	18,080 (18%)	25,658 (19%)	1,149	330
Dairy farms	354,262	310,362	278	364	10,502 (16%)	11,427(17%)	1,015	218

### Rarefaction

**Figure 1:**
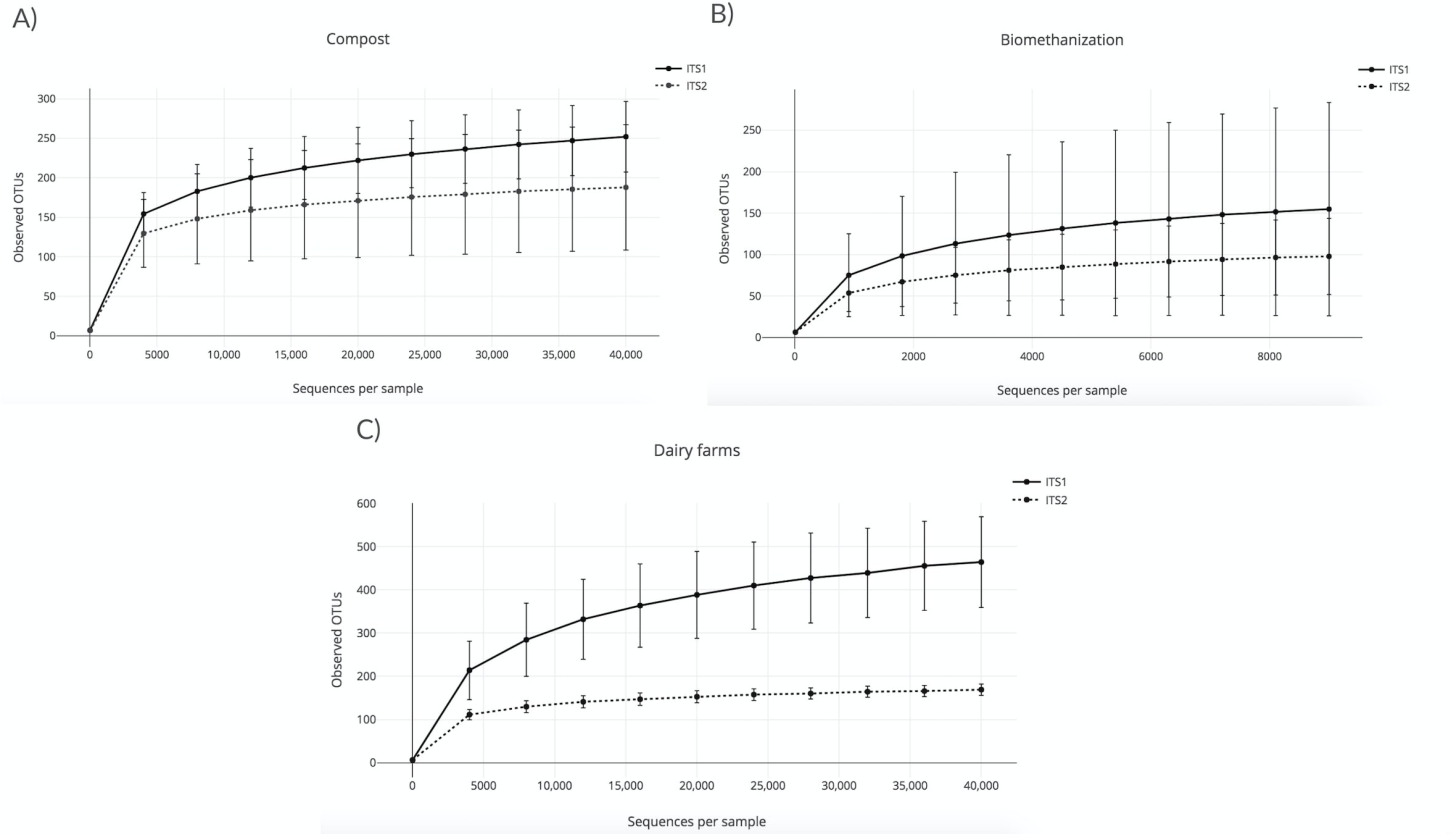
Rarefaction curves obtained from the number of observed OTUs and the sequences per sample for air samples from (A) composting sites (the plateaus of the curves started at around 5,000 sequences); (B) biomethanization (the plateaus of the curves started at around 1,500 sequences); (C) dairy farms (the plateaus of the curves started at around 5,000 sequences).

A rarefaction analysis using the observed OTU alpha diversity metric was conducted to validate the sequencing depth and confirm the effective sampling of the biological content of the aerosol samples that were collected in the three environments studied. The lowest depth was used as the sequencing depth of the rarefaction analyses. This procedure allows the rarefaction of all the samples to the same number of sequences. In other words, samples with a lower sequencing depth than the one chosen were excluded from the analyses. The higher the sequencing depth, the more likely diversity coverage is attained. In this case, the sequencing depth was 40,000 sequences per sample for compost and dairy farms (Figs. 1A and 1C), and 9,000 sequences for biomethanization (Fig. 1B). All the samples were included in the analyses, except the outdoor controls due to low sequence numbers. Outdoor controls are samples taken outside the facilities visited. The points shown in Figs. 1A (compost) and 1B (biomethanization) were calculated using ten randomly selected values from 10 to 40,000 sequences. Points shown in Fig. 1C (dairy farms) were calculated similarly but with values selected from 10 to 9000 sequences. The corresponding number of OTUs observed for each of these values was recorded for all samples. The average number of OTUs observed with the standard deviation was calculated for each one of the ten values. To analyze the rarefaction data, samples were grouped according to the barcode used. The plateaus of the curves in Fig. 1 indicate efficient coverage of the fungal diversity with ITS1 and ITS2, as no more OTUs were detected, even with greater numbers of sequences per sample. We analyzed with the chao1 richness estimator, and the conclusions were the same as the observed OTUs index.

### Alpha diversity

Alpha diversity was measured using Chao1, Shannon and Simpson. The diversity measures were obtained using the alpha diversity QIIME script. Table 3 presents a summary of the alpha diversity measures with the systematic comparison of ITS1 and ITS2 barcodes in the three environments studied. The ITS1 barcode produced significantly more richness per sample compared to the ITS2 barcode for composting (domestic *P* = 0.0009; animal *P* = 0.0006) and biomethanization (BF1 *P* = 0.001; BF2 *P* = 0.0007). The estimated richness was also higher when targeting ITS1 compared to ITS2 in the five dairy farms. However, no statistics could be calculated because there was only a single value for each dairy farm. Overall, the ITS1 barcode produced higher values of richness and diversity than ITS2 in the three environments.

**Table 3:** Alpha diversity analysis comparing data obtained from targeting ITS1 and ITS2 barcodes in aerosol samples from three environments. The numbers represent the mean values with the standard deviation for each group of samples. When the standard deviation is not shown, its value is zero (same diversity values). The highest values obtained from the comparisons between ITS1 and ITS2 are highlighted in bold type.

		Chao1		Shannon		Simpson	
		**ITS1**	**ITS2**	**ITS1**	**ITS2**	**ITS1**	**ITS2**
Compost	**Domestic (*n* = 25)**	292 ± 83^***^	214 ± 101	5 ± 0.5^***^	4 ± 1	0.9^****^	0.8
	**Animal (*n* = 25)**	289 ± 64^***^	214 ± 64	5 ± 0.1^***^	4.5 ± 0.5	0.9 ± 0.1	0.9
Biomethanization	**BF1 (*n* = 16)**	161 ± 13^**^	119 ± 54	4 ± 0.7^***^	3.8 ± 0.4	0.8	0.8
	**BF2 (*n* = 16)**	273 ± 73^***^	117 ± 48	4 ± 1	4 ± 0.7	0.9	0.9
Dairy farms	**DF (*n* = 5)**	**549 ± 96**	188 ± 12	4.3	**4.6**	0.8	0.8

**Notes.**

The asterisk (*) indicates the statistical significance of the Mann–Whitney *U* test (ns *P* > 0.05; ^∗^*P* ≤ 0.05 ^∗∗^*P* ≤ 0.01; ^∗∗∗^*P* ≤ 0.001; ^∗∗∗∗^*P* ≤ 0.0001).

### Multivariate analysis

**Figure 2:**
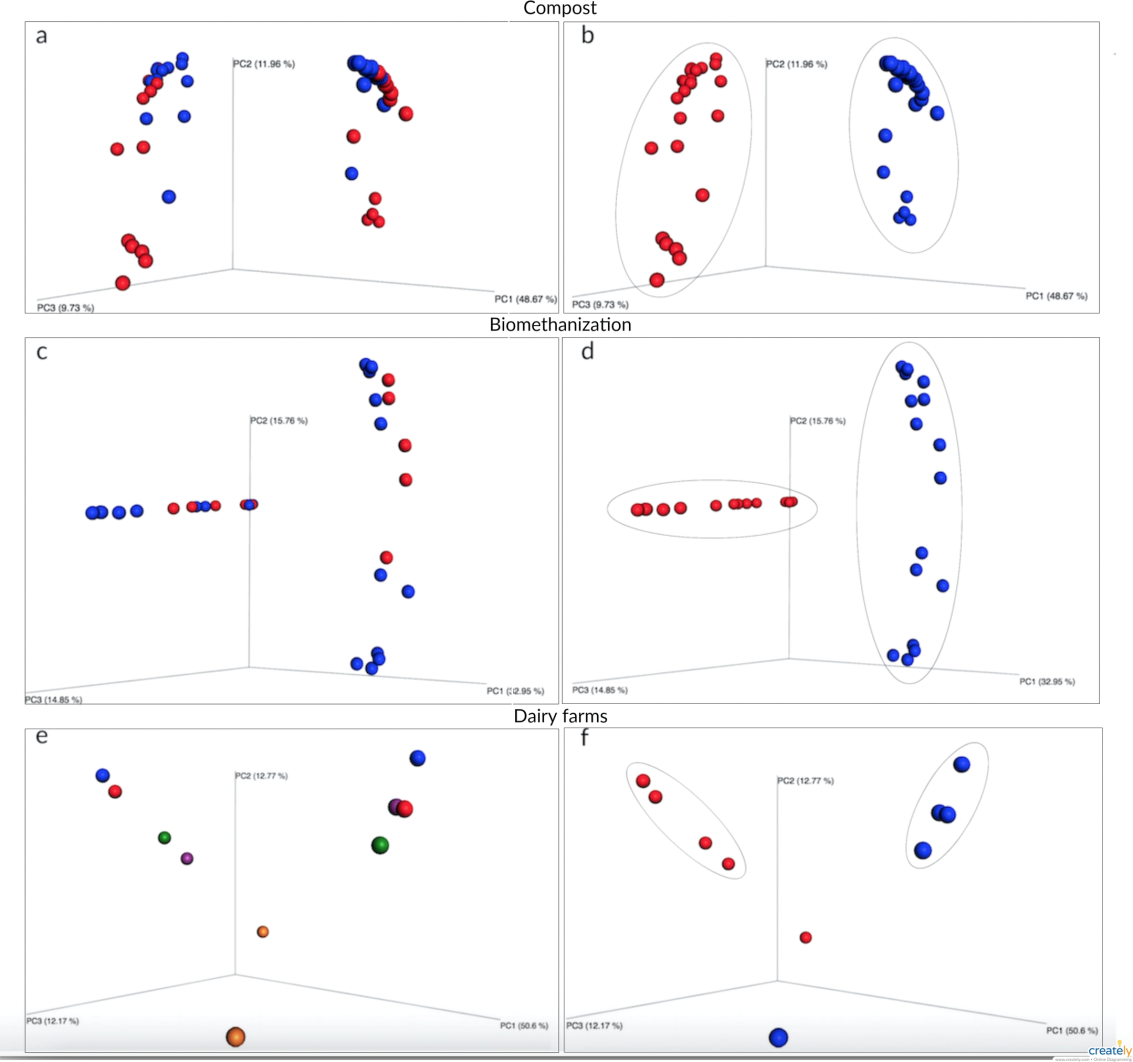
Principal coordinates analysis of air samples collected from composting sites (A and B), biomethanization facilities (C and D), and dairy farms (E and F). The PCoA plots were generated using the Bray–Curtis dissimilarity measure to calculate the distances between samples. A, C, and E show samples colored according to the type of bioaerosol source (a: domestic in blue and animal in red; c: BF1 in blue and BF2 in red; e: DF1 in blue, DF2 in orange, DF3 in red, DF4 in green, DF5 in purple). B, D and F show samples colored according to the barcode used (ITS1 in blue and ITS2 in red).

An ecological analysis was conducted to determine the strongest variable to predict change in fungal communities. The variables tested are: environmental factors and choice of barcode. One common technique used to determine the more influential variable relies on the creation of a (dis)similarity matrix to calculate the distances between samples. In this case, the Bray-Curtis dissimilarity measure was used to try and explain community variation. Figure 2 shows the three principal coordinate axes capturing more than 70% of the variation for compost (Figs. 2A and 2B), more than 63% for biomethanization (Figs. 2C and 2D), and more than 76% for dairy farms (Figs. 2E and 2F). Samples were coloured according to two variables (choice of barcode and environmental factor) to better visualize sample clustering. Samples plotted closer to one another are more similar than those ordinated further away. In each of the three environments, the choice of barcode consistently led to the best sample clustering (compost *P* = 0.001; biomethanization *P* = 0.001; dairy farms *P* = 0.007; Figs. 2B, 2D, 2F, respectively). When the environmental factor variable was used, samples were randomly dispersed with no particular colour grouping (compost *P* = 0.08; biomethanization *P* = 0.22; dairy farms *P* = 0.98; Figs. 2A, 2C, 2E, respectively). Across all three environments, the strongest predictor of fungal composition in samples was the choice of barcode used. This was a stronger predictor than the potential fungal sources present during air sampling.

**Figure 3:**
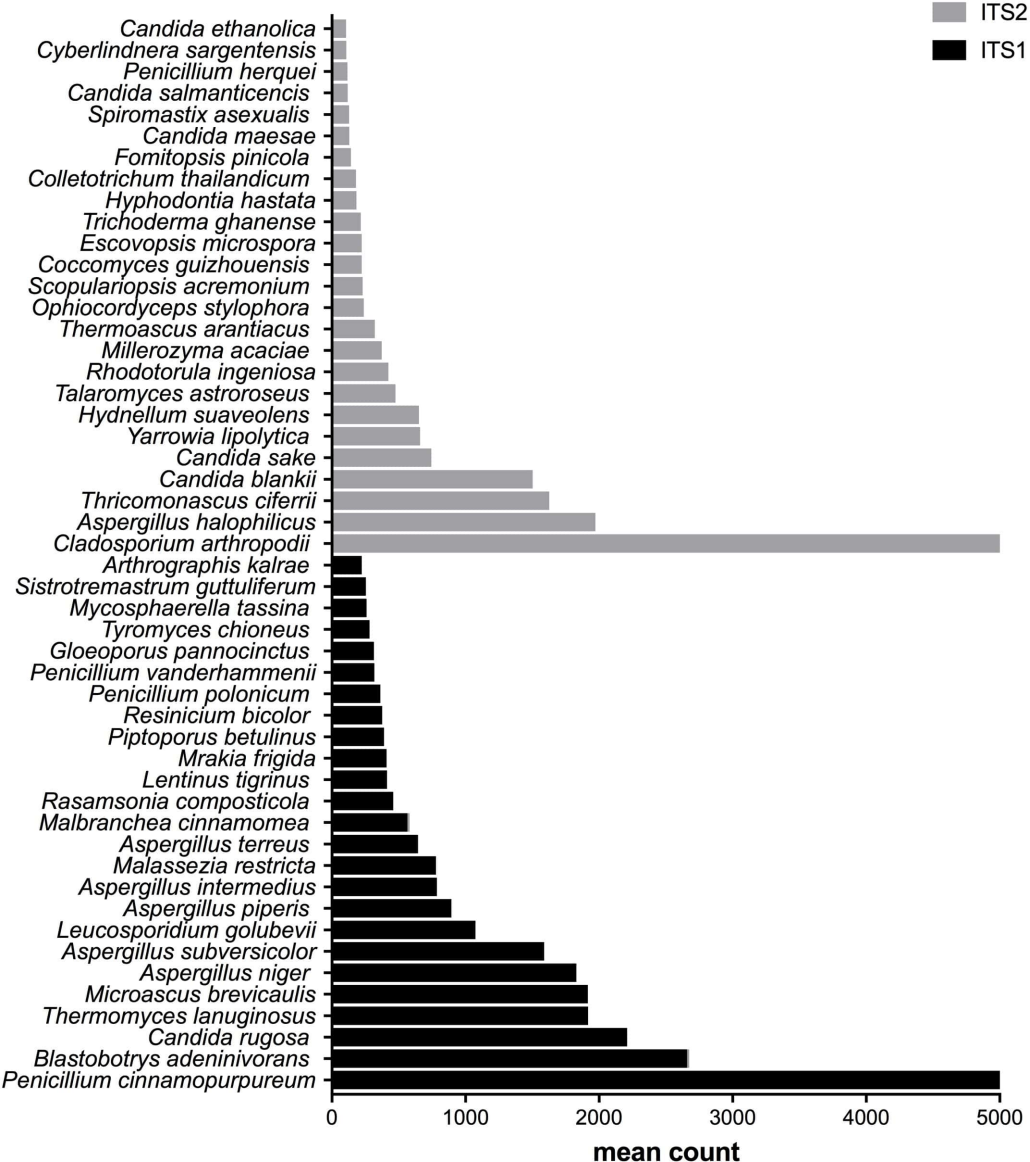
Fungal species with statistically significant differential abundances across compost samples targeting either ITS1 or ITS2 barcodes. From the bottom to the top: the first 25 species were the most abundant with ITS1 and the last 25 were more abundant with ITS2.

**Figure 4:**
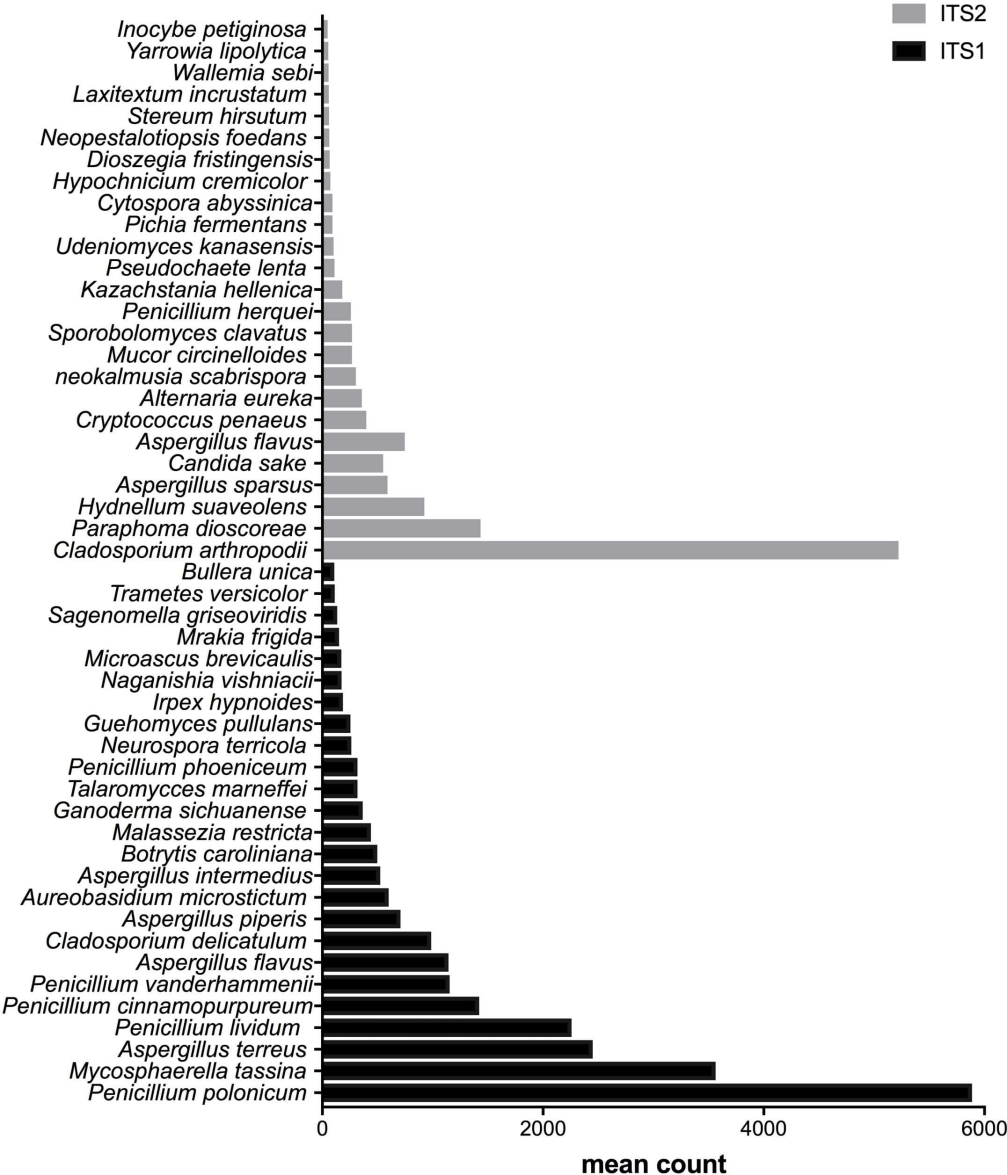
Fungal species with statistically significant differential abundances across biomethanization samples targeting either ITS1 or ITS2 barcodes. From the bottom to the top: the first 25 species were the most abundant with ITS1 and the last 25 were more abundant with ITS2.

**Figure 5:**
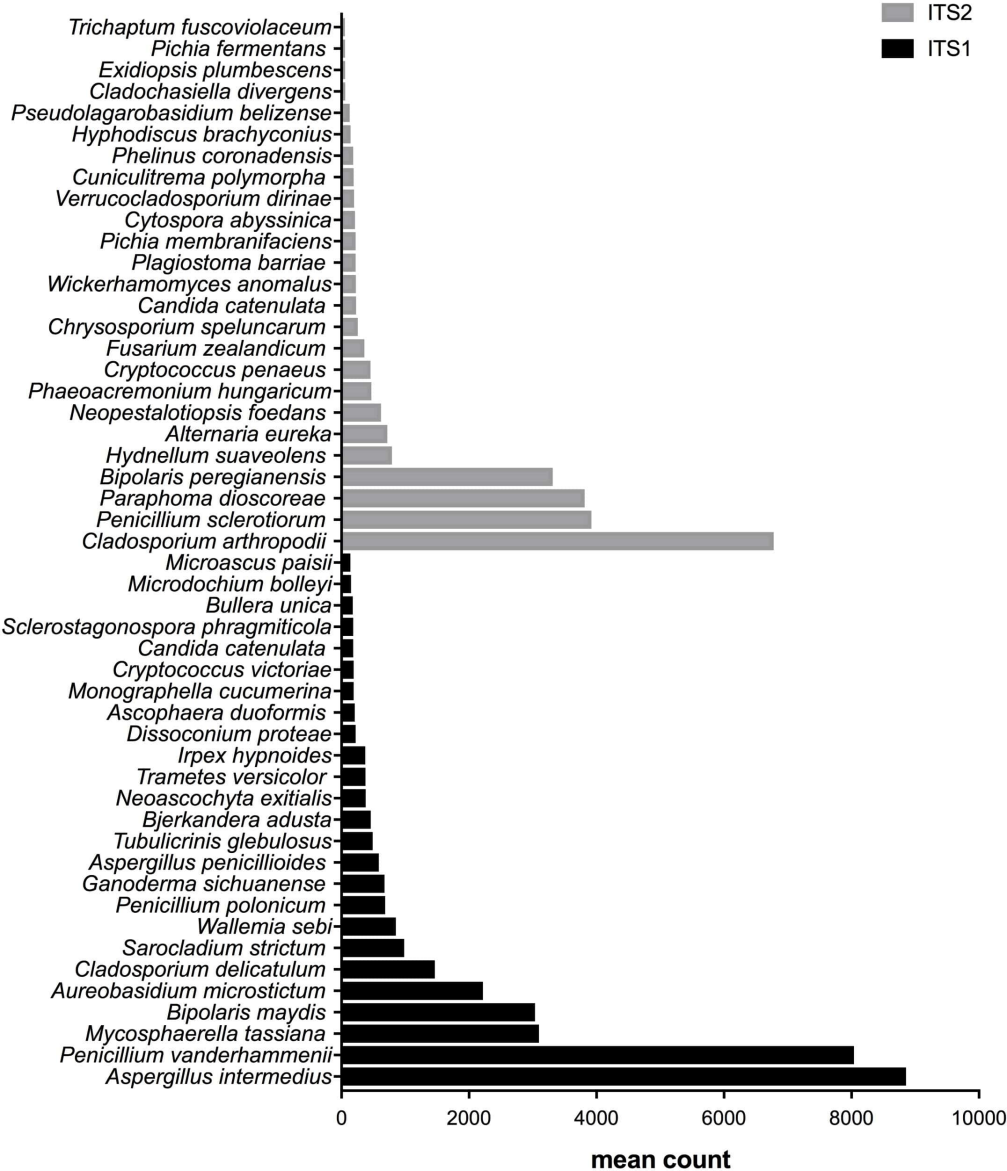
Fungal species with statistically significant differential abundances across dairy farm samples targeting either ITS1 or ITS2 barcodes. From the bottom to the top: the first 25 species were the most abundant with ITS1 and the last 25 were more abundant with ITS2.

### Differential abundances of species

After measuring the fungal community variation across samples, the next step was to try to identify species that had significantly different abundances depending on the barcode used. All the OTUs presented in Figs. 3 to 5 have large differences in mean counts between ITS1 and ITS2. However, these are not exhaustive lists of the OTUs with differential abundances. The complete outputs of the differential abundance analyses are presented in Datasets S1 to S3 for compost, biomethanization and dairy farms, respectively. The correction by the Benjamini–Hochberg FDR procedure for multiple comparisons was used for *P*-value correction. Table 4 presents the range of p-values for the 25 first differentially abundant OTUs when targeting ITS1 or ITS2 in the three environment. The most striking example in the list compiled from the compost samples is *Penicillium cinnamopurpureum,* with a mean count of 5,000 sequences across the ITS1 group and fewer than 10 sequences in the ITS2 group. Similarly, *Cladosporium arthropodii* was present with a mean count of 5,000 sequences in ITS2 and less than 5 sequences in ITS1. Surprisingly, *Cladosporium arthropodii* was also the most differentially abundant species in ITS2 samples from biomethanization facilities and dairy farms with mean counts of 5,800 sequences and more than 7,000 sequences, respectively. For ITS1, the most differentially abundant species in biomethanization samples was *Penicillium polonicum* with a mean count of 5,900 sequences and *Aspergillus intermedius* in dairy farms with a mean count of more than 8,000 sequences. These species were not detected by ITS2. In the three environments studied, the differential abundances of the 50 species were obvious as there were considerable margins in the sequence counts between ITS1 and ITS2. *Penicillium polonicum, Mycosphaerella tassina, Penicillium vanderhammenii,* and *Apergillus intermedius* were consistently more abundant in the ITS1 group and either under or not represented in the ITS2 group in all three environments. The same observation was made for *Aspergillus terreus*, *Penicillium cinnamopurpureum, Cladosporium delicatum, Aspergillus piperis, Aureobasidium microsticum, Malassezia restricta, Ganoderma sichuanense, Irpex hypnoides, Microascus brevicaulis, Mrakia frigida, Trametes versicolor* and *Bullera unica* in two of the three environments studied. Similarly, *Hydnellum suaveolens, Cryptococcus penaeus* and *Penicillium herquei* were consistently more represented by ITS2 compared to ITS1 in all three environments. This was also the case for *Paraphoma dioscoreae, Candida sake, Alternaria eureka, Pichia fermentans, Cytospora abyssinica, Neopestalotiopsis foedans* and *Yarro wialipolytica* in two of the three environments.

### Taxonomic analyses

Five classes of fungi consisting of Dothideomycetes, Eurotiomycetes, Saccharomycetes, Sordariomycetes and Agaricomycetes, were the most abundant classes in compost, representing 90% of the total relative abundance (Fig. S1). One notable difference between ITS1 and ITS2 was observed for Saccharomycetes, which was 2.5 times more abundant for ITS2 (20%) compared to ITS1 (8%). Other distinguished differences were observed in the less abundant classes, Wallemiomycetes, Exobasidiomycetes and Taphrinomycetes, which were detected only by ITS1 and Glomeromycetes, Tritirachiomycetes, Mucoromycotina, Rozellomycota and Lecanoromycetes were detected only by ITS2. In biomethanization facilities/samples, four out of 14 classes represented 90% of the total relative abundance (Eurotiomycetes, Dothideomycetes, Sordariomycetes and Agaricomycetes). Saccharomycetes were five times more abundant for ITS2 compared to ITS1. Wallemiomycetes, Exobasidiomycetes, Ustilaginomycotina and Cystobasidiomycetes were specific to ITS1 and Mucoromycotina was specific to ITS2 (Fig. S2). Similarly, four of the 14 classes that were present in dairy farm samples accounted for more than 90% of the relative abundance (Eurotiomycetes, Dothideomycetes, Sordariomycetes and Agaricomycetes). Wallemiomycetes, Exobasidiomycetes, Ustilaginomycotina and Microbotryomycetes were only present in ITS1. Lecanoromycetes and Ciliophora were specific to ITS2 (Fig. S3). Similar to compost and biomethanization facilities, the only major difference between ITS1 and ITS2 in dairy farms was the abundance of Saccharomycetes (four times more abundant in ITS2). The conclusions were the same when samples were compared according to environmental factors rather than which barcode was used (animal vs domestic for compost; BF1 vs BF2 for biomethanization; DF1 to DF5 for dairy farms; Figs. S1 to S3, respectively). Other notable differences were that Wallemiomycetes and Exobasidiomycetes were consistently only detected by ITS1 in the three environments. Lecanoromycetes was consistently present only in ITS2 across samples from the three environments. Furthermore, unidentified sequences of plants were found to be exclusive to ITS2.

**Table 4:** The statistical significance of the first 25 OTUs that were differentially abundant when targeting ITS1 and ITS2.

Type of environment	Range of *P*-values	
	**ITS1**	**ITS2**
Compost	0.0001–0.0000004	0.0008–0.000004
Biomethanization	0.004–0.0003	0.0002–0.00003
Dairy Farms	0.04–0.008	0.05–0.003

### Culture method vs HTS

The diversity of fungi identified using the culture method was compared with the fungal diversity identified using HTS at the genus level. Table 5 shows a summary of this comparison in biomethanization samples. Culture methods were able to detect six fungal genera. For HTS, the first 20 most abundant genera were considered. They represented 81% and 84% of the total relative abundance of ITS1 and ITS2, respectively. The most abundant genera were the same using both methods (*Penicillium, Aspergillus, Cladosporium* and *Talaromyces*). Although *Phialocephala* is shown to be present only in the profile obtained from the culture method, it was also detected by ITS1 and ITS2 (but not present in the top 20). The diversity profile obtained by the culture method in dairy farms was more exhaustive compared to biomethanization facilities (Table 6). For HTS, the top 20 most abundant genera accounted for 67% and 90% of the total relative abundance for ITS1 and ITS2, respectively. As in biomethanization samples, only four genera were detected by both approaches in dairy farms: *Penicillium*, *Cladosporium*, *Bipolaris* and *Fusarium.* However, some genera were shared only between ITS1 and culture (*Sarocladium* and *Aspergillus*) and between ITS2 and culture (*Wickerhamomyces* and *Alternaria*). The HTS profile of fungal genera is much more exhaustive than what is shown on the lists in Tables 5 and 6. Of the fungal genera that were isolated using culture techniques, three (*Hyphopichia, Gibellulopsis* and *Myceliophthora)* were not detected by HTS.

**Table 5:** Comparison of the 20 most abundant fungal genera identified by HTS targeting ITS1 and ITS2 barcodes, and the fungal genera identified by culture in aerosol samples collected in two biomethanization facilities. The taxa in **bold** and ***underlined***are shared between the three columns (ITS1, ITS2 and culture). Taxa in **bold** are shared between one of the two HTS columns (ITS1 or ITS2) and culture.

HTS		
ITS1 (% relative abundance)	ITS2 (% relative abundance)	Culture (% relative abundance)
***Penicillium (41.4)***	***Penicillium (22)***	***Penicillium (65.5)***
***Aspergillus (13.8)***	***Cladosporium (19.8)***	***Aspergillus (18.9)***
*Mycosphaerella (9.9)*	***Aspergillus (18.1)***	***Cladosporium (11.1)***
***Cladosporium (3)***	***Talaromyces (4)***	*Phialocephala (1.1)*
*Aureobasidium (1.7)*	*Hydnellum (4)*	***Talaromyces (2.2)***
*Cryptococcus (1.7)*	*Paraphoma (3.8)*	***Fusarium (1.1)***
*Malassezia (1.5)*	*Candida (1.6)*	
*Botrytis (1.4)*	*Peniophora (1.6)*	
*Ganoderma (1)*	*Cryptococcus (1.2)*	
***Talaromyces (0.9)***	***Fusarium (1.1)***	
*Capnobotryella (0.7)*	*Alternaria (1.1)*	
*Neuropsora (0.7)*	*Neokalmusia (0.9)*	
*Guehomyces (0.7)*	*Geotrichum (0.9)*	
*Microascus (0.5)*	*Sporobolomyces (0.7)*	
*Irpex (0.5)*	*Mucor (0.7)*	
*Naganishia (0.5)*	*Neurospora (0.5)*	
*Sagenomella (0.4)*	*Bullera (0.5)*	
*Sporobolomyces (0.4)*	*Kazachstania (0.5)*	
*Mrakia (0.4)*	*Microascus (0.4)*	
*Candida (0.3)*	*Udeniomyces (0.3)*	

**Notes.**

* The relative abundance of the HTS data represents the relative abundance of the total taxa detected (not only the top 20). For ITS1, the relative abundance of the top 20 fungal genera represents 81.4% of the total genera identified. For ITS2, the top 20 fungal genera represent 83.7% of the total genera identified.

**Table 6:** Comparison of the 20 most abundant fungal genera identified by HTS targeting ITS1 and ITS2 barcodes, and the fungal genera identified by culture in aerosol samples collected at five dairy farms. The taxa in **bold** and ***underlined***are shared between the three columns (ITS1, ITS2 and culture). Taxa in **bold** are shared between one of the two HTS columns (ITS1 or ITS2) and culture.

HTS^*^		
ITS1 (% relative abundance)	ITS2 (% relative abundance)	Culture (% relative abundance)
***Aspergillus (19.5)***	***Penicillium (39)***	***Penicillium (39.5)***
***Penicillium (13.5)***	***Cladosporium (13.7)***	***Aspergillus (22.4)***
***Fusarium (5.6)***	*Paraphoma (9)*	***Cladosporium (10.7)***
*Mycosphaerella (4.7)*	***Bipolaris (6.7)***	*Rhodosporidium (4.6)*
***Bipolaris (4.6)***	***Fusarium (6.3)***	***Sarocladium (3.9)***
*Aureobasidium (3.4)*	*Hyphoderma (2.4)*	*Hormographiella (2.5)*
***Cladosporium (2.2)***	***Alternaria (2.2)***	*Phaeosphaeria (2.5)*
*Parastagonospora (2.2)*	*Peniophora (1.7)*	***Wickerhamomyces (1.8)***
*Capnobotryella (2.1)*	*Hydnellum (1.6)*	***Alternaria (1.8)***
***Sarocladium (1.6)***	*Neopestalotiopsis (1.3)*	*Epicoccum (1.4)*
*Wallemia (1.3)*	*Cryptococcus (1)*	*Meyerozyma (1.4)*
*Ganoderma (1)*	*Phaeoacremonium (1)*	***Bipolaris (1.4)***
*Tubulicrinis (0.8)*	*Candida (0.8)*	*Lichtheimia (1.4)*
*Neoascochyta (0.7)*	*Pichia (0.8)*	*Myceliophthora (0.7)*
*Bjerkandera (0.7)*	*Bjerkandera (0.6)*	*Gibellulopsis (0.7)*
*Irpex (0.6)*	***Wickerhamomyces (0.5)***	***Fusarium (0.7)***
*Trametes (0.6)*	*Chrysosporium (0.5)*	*Hyphopichia (0.7)*
*Cryptococcus (0.6)*	*Phelinus (0.4)*	*Trichosporon (0.7)*
*Candida (0.4)*	*Phyllosticta (0.4)*	*Rhizomucor (0.7)*
*Monographella (0.4)*	*Verrucladosporium (0.4)*	*Thermomyces (0.36)*

**Notes.**

* The relative abundance of the next-generation sequencing data represents the relative abundance of the total taxa detected (not only the top 20). For ITS1, the relative abundance of the top 20 fungal genera represents 66.6% of the total genera identified. For ITS2, the top 20 fungal genera represent 90.3% of the total genera identified.

### Shotgun versus amplicon sequencing

**Figure 6:**
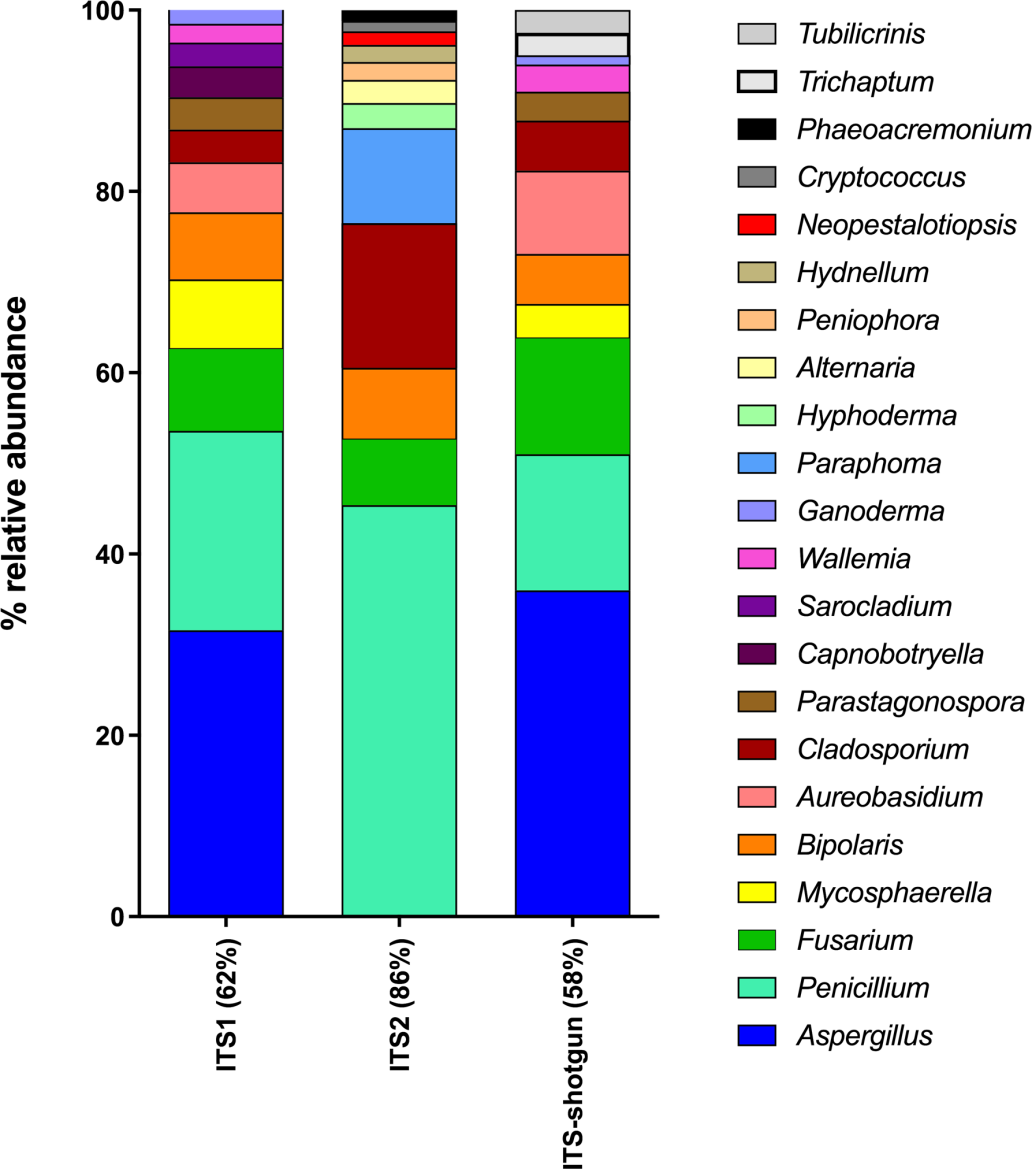
Relative abundances of fungal genera detected in dairy farms by shotgun and amplicon-based (ITS1 and ITS2) HTS. The whole ITS region (ITS1-5.8S-ITS2) was extracted from the shotgun metagenomes (ITS-shotgun) and relative abundance was recalculated based on the representative number of sequences.

Five air samples collected from five dairy farms yielded 101,652,459 high-quality metagenome sequences. Only 2,338,007 of these sequences were of fungal origin, representing 2.3% of the total sequences. Interestingly, the four most abundant classes of fungi detected using shotgun metagenomics (Dothideomycetes 39%; Eurotiomycetes 18%; Agaricomycetes 15%; Sordariomycetes 11%) correspond to the most abundant classes detected in ITS1 and ITS2 sequences. However, some fungal classes could only be recovered from metagenomes (Mixiomycetes 4%; Geoglossomycetes 2%; Orbiliomycetes 1%) and some were retrieved only from amplicon-based sequencing (Ustilaginomycotina, ITS1; Microbotryomycetes, ITS1; Ciliophora, ITS2; Leotiomycetes, ITS1 and ITS2). One of the most striking differences between shotgun and amplicon-based sequencing lies is the relative abundance of unidentified fungi, which was 10% for metagenomes and less than 2% for amplicon-based sequences. Metagenome sequences were blasted against the ITS sequences in the UNITE database in order to extract the sequences corresponding to the whole ITS region from the shotgun sequencing. Then, the taxonomic identification of these sequences were compared to those obtained by the amplicon-based (ITS1 and ITS2) HTS approach (Fig. S4). Some differences were observed in the classes identified from ITS shotgun sequences and the ITS1 and ITS2 sequences. For instance, Agaricomycetes and Pezizomycetes were significantly less abundant in the amplicon-based sequences compared to the shotgun metagenomes. In contrast, Dothideomycetes, Sordariomycetes, and Eurotiomycetes were more common in ITS1 and ITS2 sequences compared to ITS shotgun sequences. To more thoroughly examine the relationship between ITS1, ITS2 and ITS retrieved from shotgun sequencing when identifying fungal genera, we generated a taxonomic profile based on genus comparing the three components (Fig. 6). For more effective visualization, we considered only the genera that represent more than 1% of the total relative abundance for each component represented in Fig. 6. For ITS2, the genera that make up more than 1% of the total relative abundance represented 86% of the total relative abundance when combined. However, for ITS1 and ITS-shotgun, the genera that make up more than 1% of the total relative abundance represented only 62% and 58% of the abundance, respectively. This means that a more diverse genus profile is expected in ITS1 and ITS-shotgun sequences that comprise less than 1% of the total abundance. The genera identified by ITS shotgun sequences had more similarities with the ITS1 profile compared to ITS2. A striking example is the absence of *Aspergillus* from the ITS2 profile while it was present in more than 25% of ITS1 and ITS-shotgun sequences. Also, *Cladosporium* was significantly more abundant in ITS2 sequences compared to those of ITS1 and ITS-shotgun. As expected, some taxa were only detected using ITS-shotgun approach, like *Trichaptum* and *Tubilicrinis*. These genera are a part of the class Agaricomycetes, and were statistically more abundant in the metagenomes compared to the amplicons.

## Discussion

The samples used in this study are a part of published work in three different publications assessing workers’ exposure in composting sites, biomethanization facilities, and dairy farms (Mbareche et al., 2017; Mbareche et al., 2018a; Mbareche et al., 2018b; Mbareche et al., 2019a).

The internal transcribed spacer (ITS) region has been proposed as a standard genetic marker for fungi (Schoch et al., 2012). The amplicon-based HTS approach relies on the use of one of the two sub regions (ITS1 and ITS2). A comprehensive investigation validating their value as DNA barcodes in different environments is necessary for building effective strategies for characterizing fungal diversity. In this large-scale study, the systematic comparison of ITS1 and ITS2 barcodes in three different environments led to consistent results in regards to fungal diversity in bioaerosols.

In terms of sequence length, ITS2 had longer sequences than ITS1 throughout all samples from the three environments. Because longer amplicons represent a challenge for PCR amplification and DNA sequencing, this observation implies that ITS2 is more difficult to amplify than ITS1. This difference in length may be due to the fact that the 5.8S rRNA gene is included in the primers amplifying ITS2 in this study. It has been previously demonstrated that long ITS barcodes may affect amplification and sequencing (Lindahl et al., 2013; Tedersoo et al., 2015b). Furthermore, sequence length may have a strong inverse correlation with abundance recovery (Ihrmark et al., 2012). Relatively longer reads are subject to low quality at the end of the sequences, which may cause problems during paired-end ligation. The percentages of singletons were comparable between ITS1 and ITS2 across samples from all three environments. Neither barcode outperformed the other in terms of generating singletons. The importance of eliminating singletons in HTS analyses is best described with Tolstoy’s rule which states that most unique sequences are bad: ≪ *If most bases are good, most unique sequences are bad, because good reads are all alike, but every bad read is bad in its own way* ≫.

The species diversity measurement was introduced by Whittaker and defined as the number of species and their proportions within one sample (Whittaker, 1972). Different alpha diversity measures have been proposed and the choice of measure depends on the context of the study. To help make informed choices, a list of indexes and explanations on how these measures are used is presented in Magurran and McGill’s book (Magurran & McGill, 2011). Alpha diversity metrics indicate that ITS1 was consistently able to recover more OTUs and better estimate richness compared to ITS2 in all three environments. Some exceptions were noted for species diversity measurements, as ITS2 outperformed ITS1 in a few samples. However, only one sample was used for comparison in each dairy farm, which may explain the overall contradictory observations. The diversity measurements are known to perform better when a higher number of samples is used for making comparisons (Veech & Crist, 2010; Jost, 2006; Jost, 2007; Jost et al., 2010; Moreno & Rodrìguez, 2010). The authors also looked at the correlation between richness/diversity estimates between ITS1 and ITS2, and no particular correlation could be observed.

The multivariate analyses coupled with the PERMANOVA test provided robust analyses demonstrating the statistical significance of sample clusters using distance matrices. The fact that the two analyses yielded the same conclusions about the clusters formed by the samples confirms their combined usefulness as tools to visualize and measure sample clustering. The overall analyses allow the study of variables that may explain community composition. In this work, the choice of barcode was the principal factor responsible for detecting trends in the fungal composition of samples from compost, biomethanization facilities and dairy farms. These observations argue against the notion that environmental factors (e.g., source of bioaerosols) are the main variables that influence fungal composition, as previously described (Mbareche et al., 2017; Mbareche et al., 2018a; Mbareche et al., 2018b; Mbareche et al., 2019a; Mbareche et al., 2019b). In addition, even when all the samples were considered in one PCoA plot, the choice of the barcode was the best predicator of the microbial content of the samples. In light of these findings, researchers should be aware that the fungal diversity detected by amplicon-based HTS is highly dependent on the barcode used, and this should be considered in future discussions. Many studies have shown that sequencing methods, which include primer selection, are the strongest determinants of community profiles (Lozupone et al., 2013; Adams et al., 2015). However in this study, ITS1 and ITS2 similarly differentiate between samples from different sources, thus both methods come to the same biological interpretation if differences between samples were of interest.

The differential abundance analyses of OTUs across samples from multiple environments and grouped into ITS1 and ITS2 were essential in determining which species were affected by the choice of barcode. Certainly, differences between ITS1 and ITS2 are species-dependent. The fungal species that were consistently only found in the ITS1 list or ITS2 list in all three environments or at least two of the three environments should be examined more closely when planning a strategy to study the fungal diversity in aerosol samples. Moreover, rare species that were unique to each environment and only detected by ITS1 or ITS2 should also be taken into consideration. The complete information about the differentially abundant taxa is presented in the supporting material. Primer mismatches could explain the potential biases linked to the taxa positively influenced by the choice of either ITS1 or ITS2 (Bellemain et al., 2010; Han et al., 2013). The advantage of ITS2 for the class Saccharomycetes in the three environments, as showcased in the taxonomy analysis, could be linked to a 3′  terminal mismatch to ITS1 primers (Tedersoo et al., 2015a; Tedersoo et al., 2015b). As previously noted, the fungal specific ITS1F forward primer is known to have mismatches to some classes including Chytridiomycota, Saccharomycetes and some genera of Dothideomycetes. The presence of introns between primer sites some fungal groups of Ascomycota may also explain the difference between ITS1 and ITS2 (Perotto et al., 2000; Bhattacharya et al., 2000; Vrålstad, Myhre & Schumacher, 2002). Nilsson and collaborators have stated in a recent review that the choice of the primers dictates what fungi will be detected in the samples (Nilsson et al., 2018). The differential abundances of species between ITS1 and ITS2 could also be explained by the targeted rRNA gene copy number. This can lead to a consistent over or under representation of particular taxa (Taylor et al., 2016). Likewise, the presence/absence of fungal classes depending on the type of barcode used should be noted for future environmental studies targeting a specific class of fungi.

Another striking difference in the results from the two barcodes was that when the ITS2 barcode was used in the three environments, consistent unidentified sequences belonging to plants were detected. While this observation suggests that there is a need to design primers that are more specific to fungi, it raises the question of erroneous taxonomic classification. The quality and abundance of data in references may affect the identification of particular taxa, thus causing identification bias. The quality of sequences from public databases is unknown, and it is sometimes hard to distinguish which sequences cover only parts of ITS and which cover the entire region. It has been previously demonstrated that up to 20% of fungal sequences in databases may be erroneously designated (Nilsson et al., 2006; Bidartondo, 2008; Koljalg et al., 2013). Two good examples of reference-based biases are related to the identification of Cryptomycota and Microsporidia. These groups of fungi are not well described in ITS databases due to the fact that their descriptions relied on taxonomic studies focused on the small subunit 18S (Lazarus & James, 2014; Bass et al., 2018). Moreover, sequences designated as unidentified fungi (with ITS1 and ITS2) probably belong to early divergent fungal lineages that are underrepresented in ITS databases, which are valued for their intraspecific variability quality (Nilsson et al., 2008; Schoch et al., 2012). Finally, the recording of *Talaromyces marneffei*, a notable human pathogen that only occurs in Southeast Asia in conjunction with bamboo rat habitats, shows that some of these sequence-based identification will certainly be erroneous due to closely related species wrongly identified in databases. In this work, using ITS1 led to an overall identification success rate that was higher than that of ITS2 in the three environments studied.

Comparing the taxonomic profiles obtained by HTS and culture methods confirmed the expected biases in the determination of fungal diversity by culture. Extremely low numbers of species were identified by culture compared to HTS. The differences observed in the diversity profiles that were obtained by the two methods could be due to the fact that culture methods give an advantage to the rare biosphere. This hypothesis is supported by the conclusions made by Shade et al. (2012) regarding the complementarity of culture-dependent and culture-independent methods when studying microbial diversity. The proposition in their study is that culture-dependent methods are biased towards bacteria from the rare biosphere and give additional information to data obtained using HTS. Herein, the complementarity was demonstrated in relative abundance. In fact, three fungi were detected specifically by culture methods, and not HTS. In contrast, hundreds of fungi were identified by HTS alone. Another difference between the two methods is that the sequencing approaches also detect the extracellular and cell-fragmented fraction of bioaerosols, which may be responsible of the inflation of the taxa identified.

Additional characteristics could be responsible for the differences noted between ITS1 and ITS2 as fungal barcodes in amplicon-based HTS. A recent study used ITS sequences from UNITE database to examine the GC content of ITS1 and ITS2 sequences. The study showed that ITS1 had a significantly lower GC content than ITS2 which may give ITS1 an advantage in amplification and sequencing compared to ITS2 (Wang et al., 2014). Indeed, GC content is known to have an effect on PCR and sequencing efficiencies (McDowell, Burns & Parkes, 1998). Also, many bioinformatics features may affect diversity such as the clustering algorithms, the percentage of identity threshold and taxonomy assignment algorithms (BLASTn vs. Naïve Bayesian Classifier). In the present work, ITS1 and ITS2 were compared using the same bioinformatics tools in order to reduce any additional biases in the diversity analyses.

A new method has emerged that produces exact sequence variants (ESVs) instead of OTUs for a greater resolution than OTU-based methods. Other synonyms of ESVs are amplicon sequence variant (ASV), and zero radius OTU (ZOTU). Another way of expressing this concept is simply as an OTU defined by 100% sequence similarity. ASVs capture all biological variations present in a dataset, and ASVs inferred from a given dataset can be reproduced in future datasets and validly compared (Callahan, McMurdie & Holmes, 2017). However, ASVs method also comes with its share of limitations. Allowing 100% sequence similarity may lead to a wrong differentiation between the SNPs of the same species. In addition, the zero percent difference may give an extremely high number of ASVs in a sample, which, in return, causes the missing of the core microbiome information’s (H Mbareche, 2017, unpublished data). Thus, applying ASVs to the actual dataset led to more differences between ITS1 and ITS2 than the OTU approach.

The difference between the shotgun and amplicon-based HTS approaches in exclusively detecting some classes of fungi (mentioned in the ‘Results’ section) was expected. In fact, PCR biases related to amplicon sequencing are well known, and were previously addressed. However, shotgun metagenomes also present biases related to the low amounts numbers of overlapping shotgun sequences across the ribosomal DNA, which can make OTU assignment and taxonomic identification inaccurate (Tedersoo et al., 2015a; Tedersoo et al., 2015b; Bengtsson et al., 2012; Gomez-Alvarez, Teal & Schmidt, 2009). In other words, metagenome sequences may fall into any genomic region and not into any part of the SSU, ITS, LSU, or into the intergenic spacers (IGS) that are typically considered in studies of this kind. This may also explain the high percentage of unidentified fungi in shotgun metagenomes when compared to amplicon-based HTS in this study. Because the SSU, ITS, and LSU all include variable as well as highly conserved regions, it is difficult to accurately assign taxonomy to short fragments and to low taxonomic levels. Thus, there is a higher likelihood of erroneous taxonomic assignments when using the shotgun metagenomic HTS approach compared to the amplicon-based HTS approach. To improve taxonomic identification in metagenomes, databases should be revised to include full-length rDNA sequences originating from genomic studies and that cover all classes of fungi and closely related organisms. One reassuring observation that was made is that the class taxonomic profile remained highly comparable when ITS sequences were extracted from the metagenomes and when all metagenome sequences were considered. It confirms the usefulness of ITS region in predicting fungal diversity. In this sense, although shotgun metagenomics and amplicon-based HTS approaches resulted in equivalent taxonomic profiles for the most abundant fungal classes, there were substantial similarities between ITS region retrieved from metagenomes and ITS1-based sequencing when compared to ITS2 with consideration to the fungal genera. This makes ITS1 a more popular choice for/as the fungal barcode when limited resources are available. Although previous work has already compared amplicon-based HTS and metagenomic approaches, it was done in different environments using 16S rRNA gene (Tessler et al., 2017). This work deals with ITS region in bioaerosols, and, compared to the actual literature, it brings new information on the role of ITS1 in retrieving the same fungal diversity as shotgun metagenomic compared to targeting ITS2. Based on these results, shotgun metagenomics is a waste of financial and computational resources when its sole intention is to profile fungal taxonomy. In addition, this method presents considerable biases. However, the functional big data produced in metagenomic analyses makes this approach a promising tool for the future of fungal ecology (Sharpton, 2014).

Continuous advances in bioinformatics tools and experimental designs are necessary to further improve the usage of ITS1 and/or ITS2 as universal fungal barcodes. New PCR primers for ITS1 and ITS2 must continue to be developed and tested (Bellemain et al., 2010; Han et al., 2013; Op De Beeck et al., 2014; Tedersoo et al., 2015a; Tedersoo et al., 2015b). Advances in sequencing technology that improve the quality and length of reads may lead to the use of the full ITS region. This would thus reduce the biases related to choosing ITS1 or ITS2. Also, developing a method that combines the use of multiple reference datasets can help to identify the vast majority of the OTUs at all taxonomic levels. Finally, strategies to correct the gene copy number may help improve the abundance estimates from amplicon-based HTS targeting ITS1 and ITS2. This gene copy number correction had positive outcomes on the prokaryote 16S in HTS studies (Kembel et al., 2012; Angly et al., 2014).

One of the limitations of this study is the use of a well-defined mock community to confirm the conclusions of ITS1 and ITS2 comparison in describing the fungal diversity of aerosols. Because high diversity does not always indicate true community, including mock community analyses as a positive control would bring relevant insights into the performance of ITS1 and ITS2 in describing fungal diversity.

## Conclusion

There is no universal solution to cover all fungal taxonomic groups in fungal ecology assessments of bioaerosols. The goal of this work was to offer a guide for aerosol scientists to design studies addressing the fungal population in aerosols using molecular approaches. The samples from three different environments used for the systematic comparisons of the performances of ITS1 and ITS2 as barcodes makes this research unequivocal. The two barcodes evaluated are not perfect in identifying all species. The combination of both barcodes offers a wide prospect of the fungal aerosol population. For quick general analyses of fungal diversity and when limited financial resources are available, the authors recommend using ITS1. Finally, the culture comparison with amplicon-based sequencing showed the complementarity of both approaches in describing the most abundant taxa.

##  Supplemental Information

10.7717/peerj.8523/supp-1 Supplemental Information 1 Figure S1 to S4

10.7717/peerj.8523/supp-2 Dataset S1 Statistical differential abundances of OTUs identified by trageting ITS1 and ITS2 in composting sites

10.7717/peerj.8523/supp-3 Dataset S2 Statistical differential abundances of OTUs identified by targeting ITS1 and ITS2 in biomethanization facilities

10.7717/peerj.8523/supp-4 Dataset S3 Statistical differential abundances of OTUs identified by targeting ITS1 and ITS2 in dairy farms

## Additional Information and Declarations

### Field Study Permissions

The following information was supplied relating to field study approvals (i.e., approving body and any reference numbers): Field permits were not required as permission was granted by the appropriate landowners.

### Data Availability

The following information was supplied regarding data availability: Data is available at National Center for Biotechnology Information (NCBI) under the BioProject ID: PRJNA533145.
