# Supplementary material for: Comparison of the performance of ITS1 and ITS2 as barcodes in amplicon-based sequencing of bioaerosols
Source: PeerJ. 2020 Feb 17;8:e8523. doi: 10.7717/peerj.8523 (PMC7032056; doi:10.7717/peerj.8523)
Supplement: Supplemental Information 1 [file peerj-08-8523-s001.pdf]

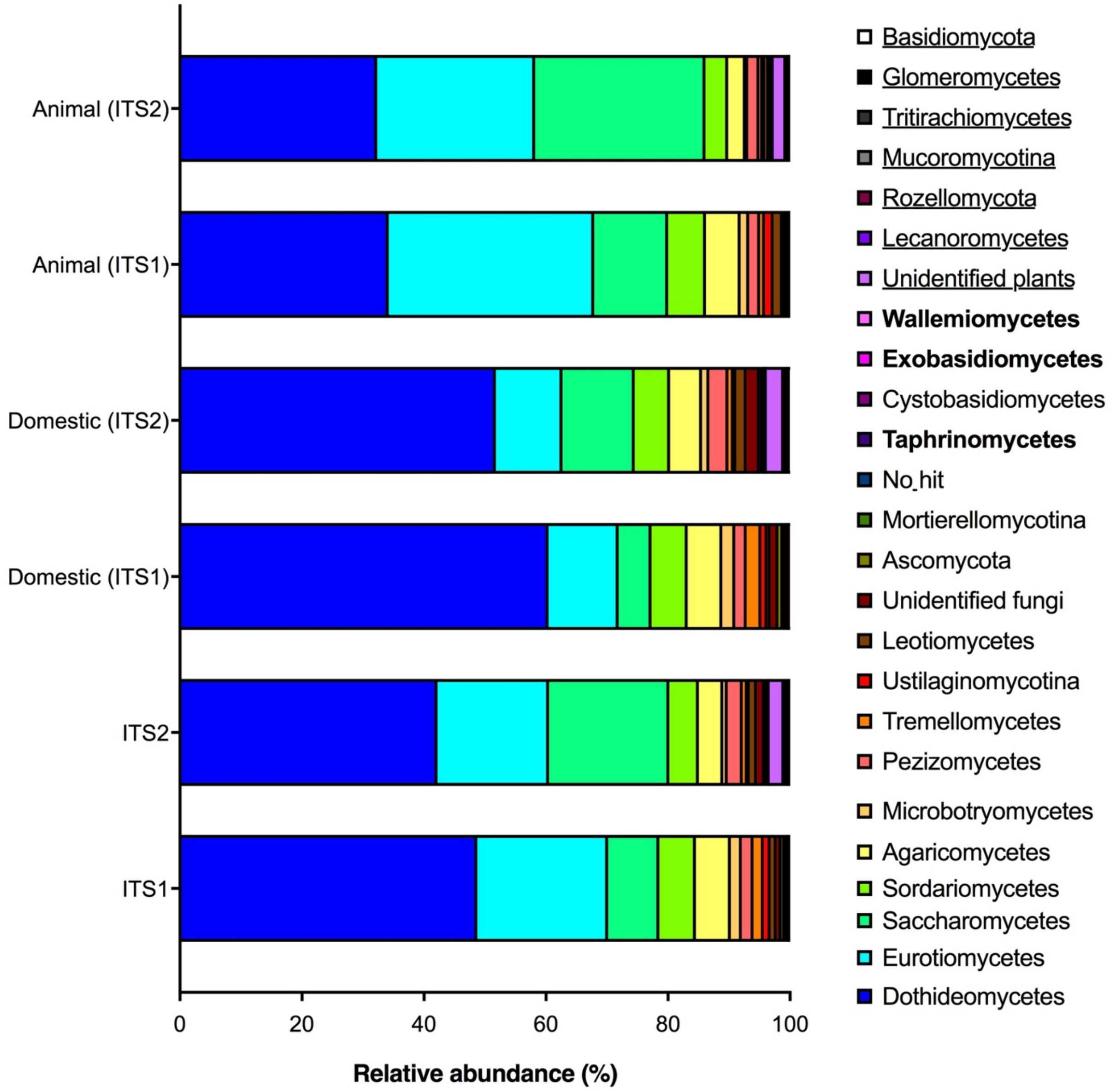

Figure S1: Relative abundances of fungal classes detected in composting sites by amplicon-based (ITS1 and ITS2) HTS method.

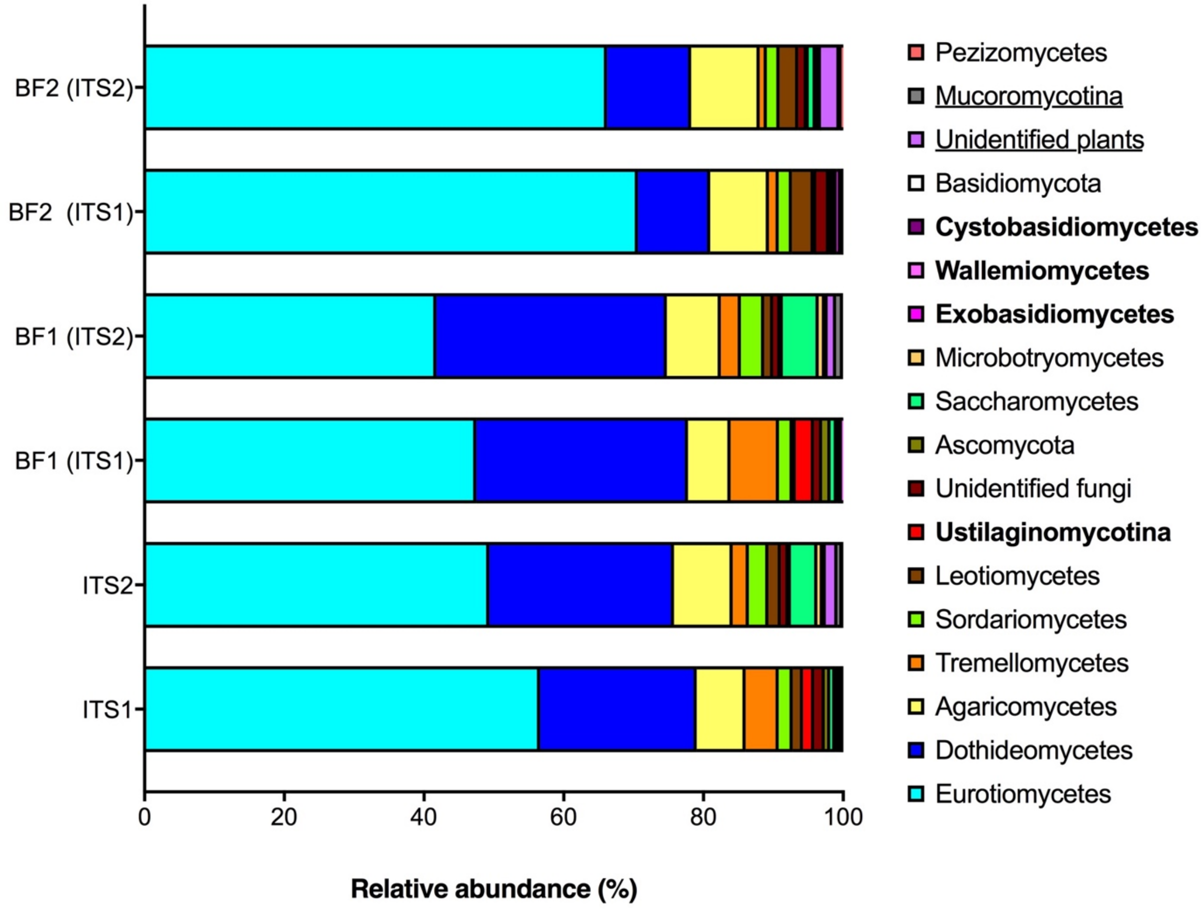

Figure S2: Relative abundances of fungal classes detected in biomethanization facilities by amplicon-based (ITS1 and ITS2) HTS method.

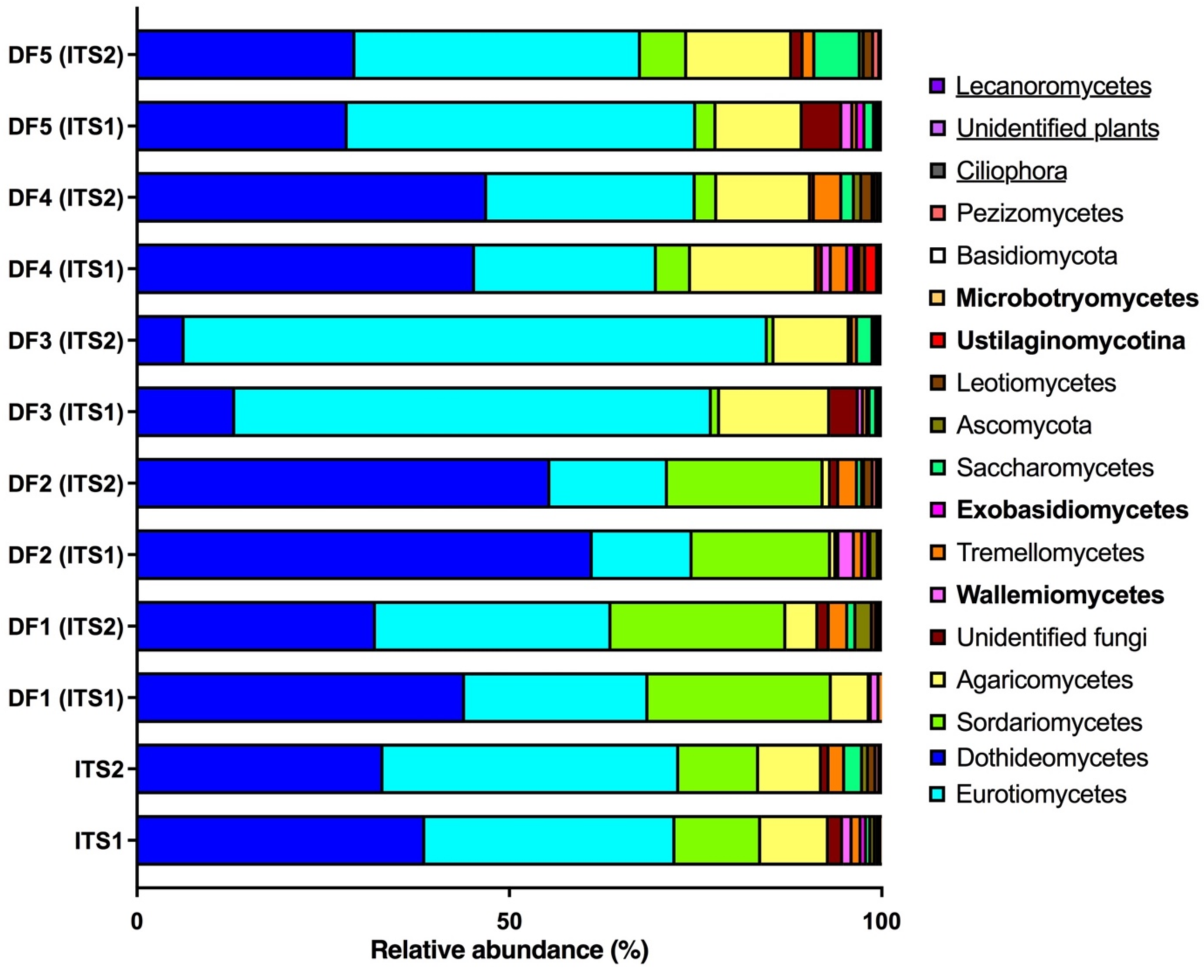

Figure S3: Relative abundances of fungal classes detected in dairy farms by amplicon-based (ITS1 and ITS2) HTS method.

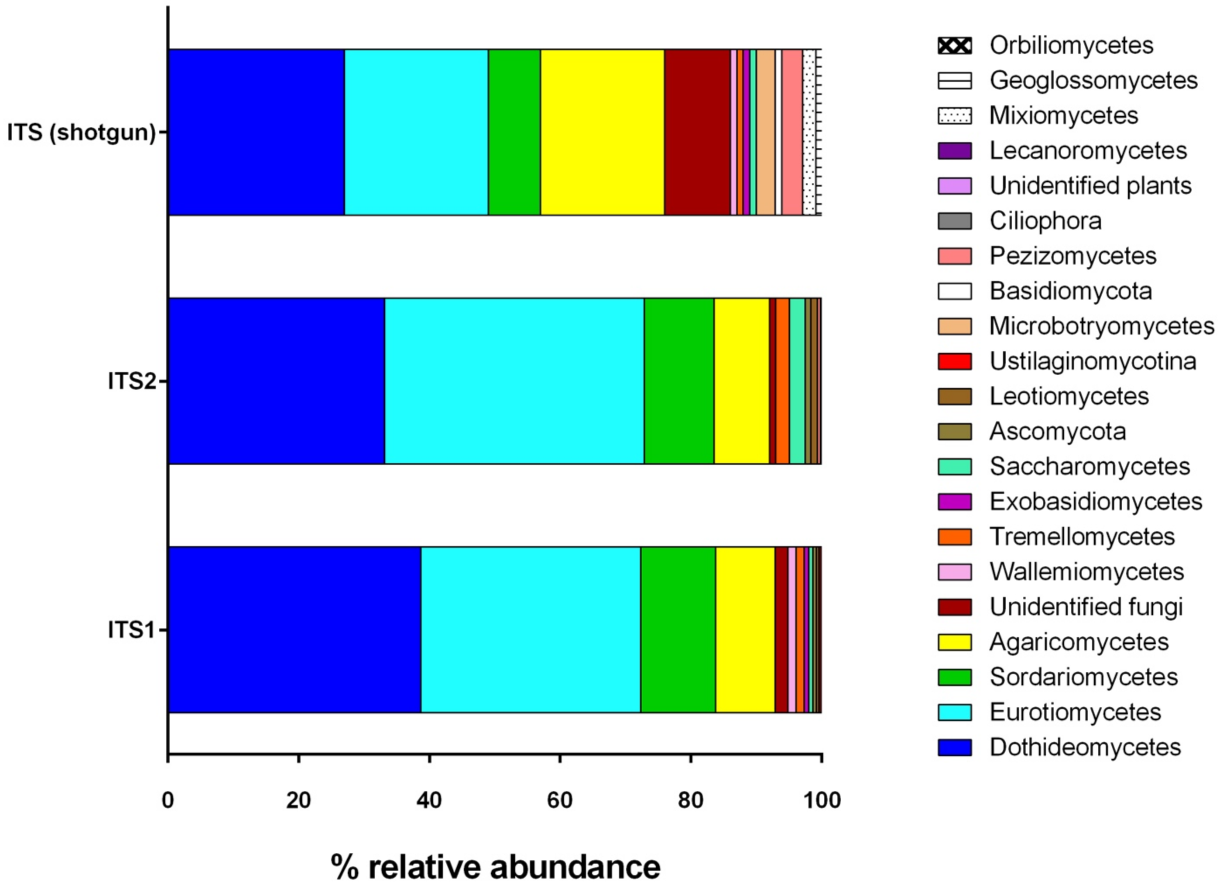

Figure S4: Relative abundances of fungal classes detected in dairy farms by shotgun and amplicon-based (ITS1 and ITS2) HTS. The whole ITS region (ITS1-5.8S-ITS2) was extracted from the shotgun metagenomes (ITS-shotgun) and relative abundance was recalculated based on the representative number of sequences.
